# Molecular Individual-Based Approach on *Triatoma brasiliensis*: Inferences on Triatomine Foci, *Trypanosoma cruzi* Natural Infection Prevalence, Parasite Diversity and Feeding Sources

**DOI:** 10.1371/journal.pntd.0004447

**Published:** 2016-02-18

**Authors:** Carlos Eduardo Almeida, Leslie Faucher, Morgane Lavina, Jane Costa, Myriam Harry

**Affiliations:** 1 Departamento de Ciências Biológicas, Faculdade de Ciências Farmacêuticas (UNESP), Araraquara, SP, Brasil; 2 UMR EGCE (Evolution, Genome, Comportment, Ecologie), CNRS-IRD-Univ. Paris-Sud, IDEEV, Université Paris-Saclay, Gif-sur-Yvette Cedex, France; 3 Programa de Pós-Graduação em Ecologia e Monitoramento Ambiental – PPGEMA, Universidade Federal da Paraíba, PB, Brasil; 4 Laboratório de Biodiversidade Entomológica, Instituto Oswaldo Cruz – Fiocruz, Rio de Janeiro, RJ, Brasil; 5 Université Paris-Sud, Orsay, France; Instituto Oswaldo Cruz, Fiocruz, BRAZIL

## Abstract

We used an individual-based molecular multisource approach to assess the epidemiological importance of *Triatoma brasiliensis* collected in distinct sites and ecotopes in Rio Grande do Norte State, Brazil. In the semi-arid zones of Brazil, this blood sucking bug is the most important vector of *Trypanosoma cruzi—*the parasite that causes Chagas disease. First, *cytochrome b* (*cytb*) and microsatellite markers were used for inferences on the genetic structure of five populations (108 bugs). Second, we determined the natural *T*. *cruzi* infection prevalence and parasite diversity in 126 bugs by amplifying a mini-exon gene from triatomine gut contents. Third, we identified the natural feeding sources of 60 *T*. *brasiliensis* by using the blood meal content via vertebrate *cytb* analysis. Demographic inferences based on *cytb* variation indicated expansion events in some sylvatic and domiciliary populations. Microsatellite results indicated gene flow between sylvatic and anthropic (domiciliary and peridomiciliary) populations, which threatens vector control efforts because sylvatic population are uncontrollable. A high natural *T*. *cruzi* infection prevalence (52–71%) and two parasite lineages were found for the sylvatic foci, in which 68% of bugs had fed on *Kerodon rupestris* (Rodentia: Caviidae), highlighting it as a potential reservoir. For peridomiciliary bugs, *Galea spixii* (Rodentia: Caviidae) was the main mammal feeding source, which may reinforce previous concerns about the potential of this animal to link the sylvatic and domiciliary *T*. *cruzi* cycles.

## Author Summary

Blood-sucking bugs are vectors of the parasite *Trypanosoma cruzi*, which causes Chagas disease. *Triatoma brasiliensis* is the main Chagas disease vector in the *Caatinga* eco-region of northeastern Brazil. We showed that *T*. *brasiliensis* exhibits gene flow between sylvatic and anthropic (domiciliary and peridomiciliary) populations. This finding represents a challenge for vector control because sylvatic foci are not reached by control activities. Bugs from sylvatic foci harbor high *T*. *cruzi* prevalence (52–71%). The potentiality that the rodent *Kerodon rupestris* may act as a parasite reservoir was suggested because bugs infected by *T*. *cruzi*, were found in the same insects that fed on this animal. Peridomestic *T*. *brasiliensis* populations exhibited high prevalence for feeding on another sylvatic rodent, *Galea spixii*, which exhibit the potential to connect *T*. *cruzi* between ecotopes. The use of an individual-based approach directed to *T*. *brasiliensis*, linking vector population genetics to host and parasite identification, may help to better understand the eco-epidemiology of Chagas.

## Introduction

Blood-sucking bugs (Triatominae, Reduviidae, Hemiptera) are vectors of the parasite *Trypanosoma cruzi* (Kinetoplastida, Trypanosomatidae), which causes Chagas disease. More than five million people are infected, and approximately 70 million live at risk [1]. An intensive and expensive Chagas Disease Control Program (PCDCh)—launched by the Ministry of Health of Brazil—was established in this country in 1975 directed primarily at *Triatoma infestans—*the vector responsible for most of cases of Chagas disease transmission in Brazil. As a result, in 2006, *T*. *cruzi* transmission by *T*. *infestans*, had been officially interrupted [2].

Once it became clear that *T*. *cruzi* was no longer actively transmitted by *T*. *infestans*, investment in entomological surveillance and control programs decreased. As a consequence, concerns are now high regarding the potential of native triatomines, such as *Triatoma brasiliensis*, for re-emerging hyperendemic Chagas disease foci in Brazil [3].

Multidisciplinary studies have demonstrated that *T*. *brasiliensis* s.l. is a species complex that forms a monophyletic group [4–6]. It includes two subspecies (*T*. *b*. *brasiliensis*; *T*. *b*. *macromelasoma)* and three species (*T*. *juazeirensis*, *T*. *melanica*, *T*. *sherlocki*). A taxonomic key has been published recently [7].

Members of the *T*. *brasiliensis* species complex are widespread in Brazil, occurring in 12 states, primarily within the *Caatinga* and *Cerrado* biomes [8–10]. Each presents distinct epidemiologic importance, morphological characteristics, natural history traits, ecological requirements, genetic characteristics and dispersal abilities [11–18].

*Triatoma b*. *brasiliensis* (hereafter, *T*. *brasiliensis*) is the best-studied member of the complex with respect to epidemiology and spatial distribution. It also has the highest prevalence of *T*. *cruzi* infection and a remarkable capacity to colonize homes. Therefore, *T*. *brasiliensis* is now the most important Chagas disease vector in the semi-arid zones of Brazil’s *Caatinga* ecoregion [17–18].

The primary difficulty with controlling *T*. *brasiliensis* is attributed to its capacity to occupy the domiciliary, peridomiciliary, and sylvatic environments [8] as it can readily re-infest treated buildings. Indeed, the actual foci for *T*. *brasiliensis* infestations are unknown: it was still unclear whether sylvatic foci are the major source of infestation or if bugs that invade domiciles are composed of domestic populations. To date, population genetic studies on *T*. *brasiliensis* have been based on a single mitochondrial gene, *cytochrome b* (*cytb*) [4,19]. But inferences using only *cytb* are limited due to its maternal inheritance and limited variation for small geographic scales.

The properties and behaviors of individuals may determine their function within the systems they compose, and therefore we used an individual-based approach directed at individuals of *T*. *brasiliensis* collected in distinct sites and ecotopes. Using this approach, we first explored the genetic structure of *T*. *brasiliensis* by using both *cytb* and microsatellites markers [20]. We then estimated the natural prevalence of *T*. *cruzi* infection and the parasite diversity in bugs and identified natural feeding sources for *T*. *brasiliensis*. Finally, we were able to make inferences about the potential *T*. *cruzi* reservoirs. We suggested that *Kerodon rupestris* (Rodentia: Caviidae) may be a potential parasite reservoir, because most sylvatic bugs had fed on this rodent and were also infected with *T*. *cruzi*, Moreover, population genetics results indicated that vector control efforts in domiciles are threatened by gene flow from the perennial sylvatic foci with high *T*. *cruzi* prevalence.

## Materials and Methods

### Sampling

Triatomines were collected in the municipality of Caicó in northeastern Brazil (between 06°23’ to 06° 41’S and 36°58’ to 37°12'W; Table 1), within the *Caatinga* ecoregion. The *Caatinga* is a mosaic of xerophytic, deciduous, semi-arid, thorny scrub. The study area chosen for insect captures was determined based on unpublished reports of public health institutions in charge of vector control, which indicated a combination of high pressure of infestation by *T*. *brasiliensis* after control activities with records of bugs infected by *T*. *cruzi* captured in domiciliary units. All investigated sites had not been sprayed in the three years prior the captures (March). All captures were conducted during the rainy season.

**Table 1:** Bug sampling: populations (labeled with letters), localities and ecotopes. Geographic coordinates and life stage are also provided.

Populations	Locality	Ecotope	Geographic coord. (S/W)	Adults	Nymphs	Total
X	São Fernando	Dom	06 23 09.1 / 37 12 09.0	1	1	2
P	São Fernando	Dom	06 23 12.6 / 37 12 08.0	1	2	3
T	São Fernando	Dom	06 23 16.1 / 37 12 04.1		1	1
R	Caicó/Sino	Dom	06 32 30.6 / 37 04 47.3	1		1
J	Caicó/downtown*	Dom	06 28 24.5 / 37 05 25.9	1	2	3
U	São Fernando	Dom	06 23 16.1 / 37 12 04.1	1	1	2
B	São João do Sabugi	Dom	06 41 58.0 / 37 10 22.6	2	41	43
N	São Fernando	Per	06 25 04.8 / 37 11 26.5		14	14
D	Caicó/Sino	Per	06 32 23.4 / 37 05 00.0	17	14	31
M	Caicó/Sino	Per	06 32 30.6 / 37 04 47.3		4	4
H	Caicó/Sino	Per	06 32 29.8 / 37 04 46.6	2	33	35
A	Caicó/EPA**	Syl-c	06 28 25.0 / 37 05 21.4	21	35	56
C	Caicó/EPA**	Syl-c	06 28 21.6 / 37 05 12.5	27	22	49
F	Santana	Syl-d	06 27 29.3 / 37 05 12.5	7	38	45
S	Solidade	Syl-d	06 28 23.6 / 37 06 12.1		8	8
Total				81	216	297

In Caicó, there is a park with lodging* for the guards. This park has its natural vegetation and fauna preserved by the armed forces and is the main Environmental Preserved Area (EPA)** close to downtown. Syl-c (conserved sylvatic), Syl-d (degraded sylvatic), Per = peridomiciliary and Dom = domiciliary.

We sampled five sites for bugs: Caicó, São Fernando, São João do Sabugi, Santana and Solidade. At each site, we collected samples in the three ecotopes where this species is found: 1) domiciles (Dom), or the indoor spaces of homes where triatomines are generally found in the crevices of mud walls, in furniture and under beds; 2) peridomiciliary areas (Per), or the areas outside but within approximately 100 m of homes where domesticated animals sleep or are maintained; and 3) sylvatic areas (Syl), or areas that are separate from peridomicilary areas and not occupied by humans. We maintain the traditional “sylvatic” terminology. However, we further divided sylvatic areas into “Syl-d”—degraded areas—where domesticated animals can be found (sites F and S; see Table 1),—and “Syl-c”—for conserved areas. These conserved areas belong to armed forces and are surrounded by huge halls. They are used only by soldiers during combat training. These areas are also protected from hunting. Domestic animals (mainly cats) are sometimes found in Syl-c, but they are removed in order to keep native fauna preserved.

In domiciliary units, bug searches were conducted as routinely employed by local vector control-surveillance staff. We investigated all ecotopes usually colonized by *T*. *brasiliensis* in peridomiciles (e. g. storerooms, henhouses, corrals, pigsties, and piles of tiles, bricks or stones) and domiciles (e.g. under stoves, beds, between spaces of roof, behind pictures fixed on the wall, in stored stuff, as food, home materials) and manually captured with tweezers all bugs observed. In sylvatic environments, similar manual methods were employed; however, insects were searched with flashlights during the night in rocky outcrops. Domiciliary and peridomiciliary populations were defined according to the villages, in which bugs from three houses were sampled. Sylvatic populations were defined according to the rocky outcrop. In the study area, these formations were ~50 square meters. We obtained permission from house owners/residents to collect insects from all homes and properties.

We identified adult vectors to species according to the taxonomic key [7,21]. Nymphs can only be identified molecularly because there are no morphological keys currently available. All the samples used in this study were also identified at the molecular level using *cytochrome b* marker (see below).

### Natural *T*. *cruzi* infection prevalence detected via optical microscopy

To identify whether insects were carrying *T*. *cruzi*-like parasites, we began with the traditional method used by local control-surveillance systems (Rio Grande do Norte state's Health Department): one fecal drop from each bug was obtained by abdominal compression, then diluted in saline solution (approximately 50 μl) and examined fresh by microscopy at 220-400X. Because starved insects do not eliminate enough fecal drops, the success of this approach depends on the insects’ nutritional status and 90 insects could be examined.

### Molecular analysis individual-based approach

The insects used for molecular studies were placed in absolute ethanol. Only adults and fifth instar nymphs (N5) were used for population genetic analyses to decrease sampling-strategic bias. Because older stages exhibit higher mobility, this strategy decreases the probability of sampling several relatives. Additionally, only populations with *N* ≥17 were used to ensure robust inferences. The resulting dataset for insect studies on population genetics was derived from 108 individuals distributed among five populations.

### Vector/parasite characterization and feeding sources

Following previous study [22], two sets of DNA extractions were performed for 126 insects with the DNeasy Tissue Kit (Qiagen): the first came from legs (DNA L) and the second from the digestive tract (DNA DT). By using DNA L, we amplified a portion of the insect *cytb* gene with the CYTB7432F/CYTBR primers [23,24], (Table 2). DNA DT was used to identify both the feeding sources, by direct sequencing *cytb* with the vertebrate-specific primers L14841/ H15149 [25] and natural *T*. *cruzi* infection, by using a pool of three primers (TCI/TCII/TC) that amplify the non-transcribed intergenic region of *T*. *cruzi* mini-exon gene. TCI is specific to the *T*. *cruzi* I strain and TCII for *T*. *cruzi* II strain, generating a fragment of 300 and 350 bp, respectively [26] (Table 2). A DNA DT from *Rhodnius prolixus* experimentally infected with *T*. *cruzi* (CL strain) was used as a positive control. The negative control was purified water. Given the putative epidemiologic threat from domiciliary vectors, the *T*. *cruzi* natural identification and characterization was also conducted on small sample sizes (N≤17).

**Table 2:** Molecular makers (*cytb* and *microsatellites*) used in this study and PCR conditions. The annealing temperature is indicated as Tm (°C). The repeat motif is given for microsatellites.

Gene/Locus	Primers (5’-3’)	References	Tm (°C)	Fragment size (bp), Motif (microsatellite)
CYTB7432F, CYTBR	F:GACG(AT)GG(AT)ATTTATTATGGATC, R:ATTACTCCTAGCTTATTAGGAATG	[23, 24]	50	458
L14841, H15149	F:AAAAAGCTTCCATCCAACATCTCAGCATGATGAAA, R:AAACTGCAGCCCCTCAGAATGATATTTGTCCTCA	[25]	50	305
TCI, TC2, TC	TC1:CCTGCAGGCACACGTGTGTGTG, TC2:GTGTCCGCCACCTCCTCCGGCCC, TC:CCCCCCTCCCAGGCCACACTG	[26]	61	350 (*T*. *cruzi I* strain), 300 (*T*. *cruzi II* strain), Depending on the strain*
Tb728	F:CTACAGCGATTTGTCTCG, R:TATTGCATCATGTTTATTGG	[20]	47	302-330pb, (GT)_2_AT(GT)_12_
Tb830	F:TGTCAGATGCATGGTGATAC, R:CATGGAAGATACCTAAACGG	[20]	60	271-288pb, (AC)_15_
Tb860	F:CGTTTTAGTAAGGAATGG, R:ATTGTGCCAAAATCAGGT	[20]	47	390-420pb, (CT)_5_ (CA)_10_(CTCA)_3_
Tb7180	F:TGACCTACCGCCACATTAC, R:AAATTTTCGATACCGCGATAG	[20]	60	207-251pb CATA)_3_(CA)_8_TA(CA)_18_(GA)_3_
Tb8124	F:GCCACTGTGTTCTCATTCC, R:TGGTGTGATGCTCAGAAGG	[20]	54	213-255pb, (CA)_18_
Tb2146	F:GCCGGTCACAATGTATCT, R:CAAAATCACTGAAAAGG	This study (KT355796)	52	146-167pb, (AC)_4_CC(AC)_4_AA(AC)_4_
Tb8102	F:CTGTCTAGGCTACTTCTTATTCTC, R:ATAAGTATTCACAGCAGAACGG	This study (KT355797)	56	255-291pb, (AC)_11_
Tb8150	F:TTGCCTAAACGGAATAATAAG, R:TTTGGAGTGGATAAGTGG	This study (KT355795)	TD: 60→50	183-197pb, (CA)_11_

*The length fragment is used to characterize the *T*. *cruzi* strain by PCR as mentioned in Materials and Methods. TD = touchdown consisting of an incremental annealing temperature decrease.

For *cytb* amplification, PCR were performed with 25 cycles (94°C 60 s, Tm 60 s, 72°C 240 s; and for *T*. *cruzi* identification with 27 cycles (94°C 60 s, Tm 30 s, 72°C 30 s) (Table 2). All amplifications were done on a thermal cycler Biometra T-1 Termoblock (Germany). The amplified products were observed in 1% agarose gel, stained with ethidium bromide. PCR products were purified using a purification method with exonuclease I (exoI) and shrimp alkaline phosphatase (sAP) to degrade excess primers and nucleotides. PCR products were mixed with 1.5 μl of phosphatase buffer, 0.19 μl of exoI, 0.45 μl of UsAP (USB, Cleveland, USA) and incubated for 1 h at 37°C. The enzymes were inactivated for 15 min at 72°C. The DNA samples were quantified on Nanodrop 2000c Spectrophotometer (Termo Fisher Scientific Inc.). All primers used and annealing temperatures for each PCR are given in Table 2. Sequence reactions were performed using Bigdye Terminator v3.1 Matrix Standard Kit (Applied Biosystem, UK) and sequences were obtained using an automatic DNA sequencer (ABI PRISM 3130 Genetic Analyzer sequencer, Applied Biosystem, UK). An initial alignment of each partial gene was conducted with the ClustalW2 algorithm [27], and manual adjustments were made using SeaView v4.3.0 [28]. To identify the feeding sources and *T*. *cruzi* strains sequences were compared to those from the GenBank database using BLAST procedures. We only selected the best hit for each search using the cut off values of 95% query coverage and 10^−100^ E -value. The name of the putative host was retained at the specific level when the sequence identity was higher than 95%, but only at the genus level when it was in the range of 75–95% especially for the cold-blooded animals because sometimes they do not have an expressive representation in GenBank.

For microsatellite genotyping, 108 adults and N5 were used, belonging to A (*N* = 22), B (*N* = 17), C (*N* = 23), D (*N* = 27) and F (*N* = 19) populations. The available primers for seven *T*. *brasiliensis* loci [20] were tested. We also revisited all the sequences with microsatellite inserts from our previous microsatellite-enriched genomic library and designed new primers for three loci (Tb8102, Tb 8150, Tb2146) using OLIGO software (version 4.0; National Biosciences Inc.). For each primer set, the optimal annealing temperature was determined using a gradient PCR (MJ Research). In total, eight microsatellite loci were used in this study, because some previously designed [20] were monomorphic for our dataset (Table 2).

PCR amplifications were performed using dye-labeled primer (Applied Biosystem) and for 40 cycles (denaturation at 94°C for 30 s, annealing for 30 s, and extension at 72°C for 30 s; Table 2). Microsatellite data were collected on an ABI Prism 3100 (Applied Biosystem) and alleles analyzed using GeneMapper (Applied Biosystem), according to previous study [20].

### Population genetic analyses

We used two procedures to confirm the specific status of *T*. *brasiliensis*. First, we applied the Automatic Barcode Gap Discovery (ABGD) method [29], using Kimura’ genetic distances, gap width X = 1, and a set of prior minimum genetic distances ranging from 0.001 to 0.1 (http://wwwabi.snv.jussieu.fr/public/abgd/abgdweb.html). Second, Bayesian inference (BI) was used to reconstruct phylogenetic relationships among unique haplotypes using MRBAYES v3.1.2 [30], with the best-fit substitution model for the data under BI through the Akaike Information Criterion (AIC) determined with jModelTest 0.1.1 [31]. We ran 2,000,000 generations using the MCMC algorithm with a burn-in period of 500,000 using a GTR+I+Γ model. The dataset was composed of the sequences obtained in this study in addition to some downloaded from GenBank for members of *T*. *brasiliensis* species complex [4,5,20]. *Triatoma sherlocki* was used as outgroup.

For *cytochrome b* analysis, the following population genetic summary statistics were calculated for the same set of data (108 N5 and adults): number of haplotypes (Nh), haplotypic diversity (Hd) (Nei, 1983), nucleotide diversity (Pi), mean number of pairwise *cytb* differences (theta) and average number of nucleotide differences (k) [32].

The evolutionary relationships among mitochondrial genotypes (genealogy) were evaluated using Network version 4.6 [33]. Tajima’s *D* [34] and Fu’s *FS* statistics [34–35] were performed to detect departure from a standard neutral model at equilibrium between mutation and genetic drift. For raw microsatellite data, possible genotyping errors due to stuttering, short allele dominance, and null alleles were tested using MICROCHECKER 2.2.3 [36]. Linkage disequilibrium was tested between all pairs of loci overall and in each population using FSTAT [37]. Deviations from Hardy Weinberg equilibrium were tested at each locus within each population using ARLEQUIN.3.0 [38], and the global test was run in GenePop [39]. Inbreeding coefficients (F_*iIS*_) were estimated using FSTAT [37].

We compared populations using F_*ST*_ pairwise estimates computed with ARLEQUIN [38]. Bonferroni correction was used throughout all levels to account for multiple testing [40]. Isolation by distance (IBD) was tested with a Mantel test for matrix correlation between pairwise genetics using distance (F_ST_/(1-F_ST_)) [41] and pairwise geographical distances with 10,000 randomizations and a reduced major axis regression (RMA) to calculate the slope in the program IBDWS version 3.23 [42].

The distributions of the expected heterozygosity for each locus and population under both the Infinite Allele Model (IAM) and the Stepwise Mutation Model (SMM) were calculated using BOTTLENECK [43]. The probability for the sign tests (p_s_) for heterozygosity excess and for p_w_ Wilcoxon test (two-tailed for H excess or deficiency) were calculated. Finally, Geneclass2 was used to select or exclude populations as the origins of individuals [44].

## Results

### Insects

Fifteen sites (Rio Grande do Norte, Brazil) were sampled: four in sylvatic areas, four in peridomiciliary and seven in domiciliary environments (Table 1). A few *T*. *pseudomaculata* (*N* = 14) and *T*. *petrochii* (*N* = 3) adults were collected and excluded from further analysis, after identification by morphology. We identified 81 adult insects as *T*. *brasiliensis* by morphology, and the remaining 216 immature insects were identified as *T*. *brasiliensis* based on our molecular analysis (see below).

### *Cytb T*. *brasiliensis* identification and genetic structure

We used 108 insects that were composed of N5 and adults in this approach. Using the ABGD (Automatic Barecode Gap Discovery) species delimitation tool, the distribution of genetic distances display two modes separated by a “barcode gap” between 0.04 and 0.07 that leads to partitions with 5 groups for all taxa of the *T*. *brasiliensis* complex analyzed. This was true for all substitution models used (p, KP or Tamura-Nei distances). All of our samples were clustered in the *T*. *brasiliensis* main group with 100% Bayesian posterior probability (S1 File).

*Cytochrome b* sequences showed 30 variable sites, including 13 singletons, resulting in 28 haplotypes for the 106 adults and N5 (Table 3). Overall, haplotype diversity was 0.89, ranging from 0.77 (peridomiciliary population D) to 0.92 (domiciliary population B). Domiciliary population B had the highest number of singletons (*N* = 7), while the sylvatic population F had the lowest (*N* = 3). The three sylvatic populations had similar haplotype diversities (population A, 0.85; C, 0.87; F, 0.90). Overall nucleotide diversity was 0.0054, ranging from 0.0051 (populations A, C and D) to 0.0069 (population B). To explore demographic population events, the Fu’s *FS* statistics were estimated (Table 3). Three populations (B, C and F) showed significant negative Fu’s *FS* values, especially the sylvatic population C (P = 0.009), suggesting expansion events. No significant departure from the standard neutral model was observed by the Tajima’s *D* values.

**Table 3:** *Triatoma brasiliensis* population genetic parameters using cytb sequences. Population labels (Pop), number of samples (*N*), variable sites (Vb), singletons (Sing), number of haplotypes (Nh), haplotype diversity ± variance (Hd), nucleotide diversity (Pi), average number of nucleotide differences (k), Fu’s FS (*FS*) and P value (p), and Tajima’s D (*D*), and p-value significance (sign).

Pop	N	Vb	Sing	Nh	Hd	Pi	k	*FS*	p	*D*	sign
A (Syl-c)	22	11	5	8	0.853±0.002	0.0051	2.398	-1.339	0.115	-0.710	no
B (Dom)	17	14	7	10	0.919±0.002	0.0069	3.191	-3.201	**0.028**	-0.877	no
C (Syl-c)	23	12	6	11	0.87±0.002	0.0051	2.379	-4.401	**0.009**	-0.929	no
D (Per)	27	14	6	9	0.775±0.005	0.0051	2.410	-1.716	0.086	-1.148	no
F (syl-d)	19	10	3	9	0.897±0.002	0.0057	2.676	-2.732	**0.043**	-0.348	no
All	108	30	13	28	0.890±0,0004	0.0054	2.551				

Syl = sylvatic, Per = peridomiciliary and Dom = domiciliary, Syl-c = conserved sylvatic, Syl-d degraded sylvatic

**Fig 1:**
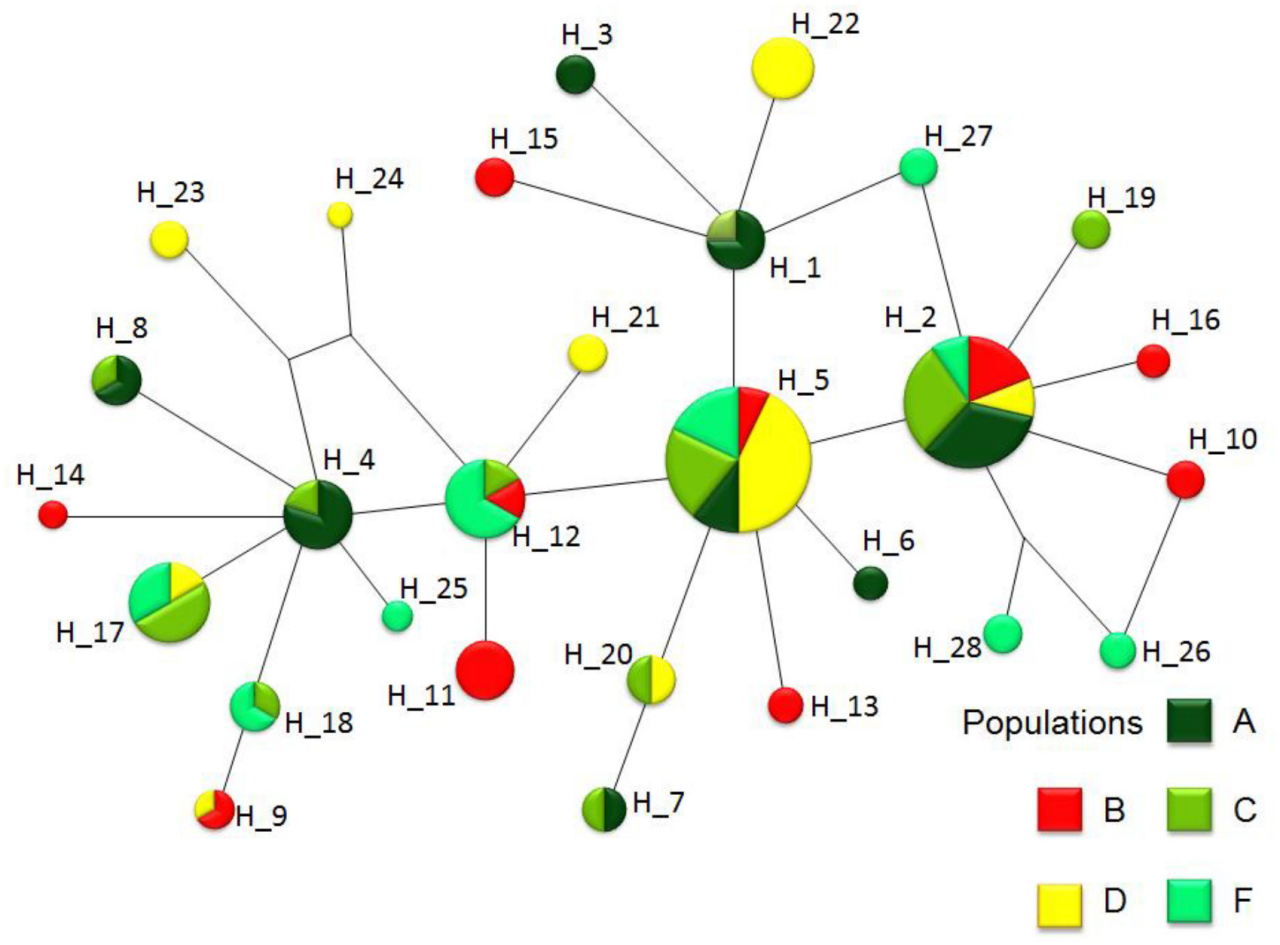
Maximum parsimony network showing the relationship among the 31 *Triatoma brasiliensis* haplotypes based on the *cytochrome b* mitochondrial gene, coded according to populations.

Eleven haplotypes were shared by at least two populations; two haplotypes had the highest frequencies (18.87%; 25.47% for H_2 and H_5, respectively) and occurred in almost all sites. Four haplotypes were shared by the sylvatic and peridomiciliary populations, while the domiciliary population shared only two haplotypes with the sylvatic populations and three with the peridomiciliary populations. The sylvatic population C shared the most haplotypes with the others; namely, H_6 and H_5 with sylvatic populations A and F, respectively. The peridomiciliary population D and domiciliary population B showed the most unique haplotypes (4 and 6, respectively). The haplotype network (Fig 1) was relatively shallow, with a maximum of four mutational steps from central haplotypes (H_2 and H_5).

### Microsatellite genotyping and genetic structure

Eight microsatellites loci were used to perform population genetic analyses (Table 4). The sylvatic population F and domiciliary population B had the lowest mean number of alleles per locus (4.33 and 5.0, respectively) and the peridomiciliary population D and sylvatic population A had the highest (6.29 and 5.86, respectively). Populations A and C had the lowest observed heterozygosity (0.45 and 0.47) and populations D and B had the highest (0.51 and 0.52). Except for population A, significant departures from Hardy-Weinberg equilibrium were found in three loci and linkage disequilibrium was detected for one (populations A, C and D) to three (population B) pair of loci. With our sampling strategy (N5 and adults), no significant heterozygote deficiency (F_*IS*_) was observed, except for population A. Under the SMM, three populations (A, C and D) showed a significant heterozygote excess with the two tests used, but this was not the case using the IAM (Table 4).

**Table 4:** *Triatoma brasiliensis* population genetic parameters using eight microsatellites. A: mean number of alleles, Ho: observed heterozygosity, He expected heterozygosity, F_IS_ index, probability for the sign tests (p_s_) for heterozygosity excess and for the two-tailed Wilcoxon test for H excess or deficiency (p_w_) for the Infinite Allele Model (IAM) and the Stepwise Mutation Model (SMM). In parentheses: ratio of number of loci with heterozygote excess to the number with heterozygote deficiency.

	N	A	Ho	He	F_IS_	IAM	SMM
A (Syl-c)	22	5.857 ± 3.288	0.44643 ± 0.19352	0.49595 ± 0.20836	**0.1018**	(4/2) p_s_ = 0.23 p_w_ = 0.56	(6/0) p_s_ = **0.004** p_w_ = **0.015**
B (Dom)	17	5.00 ± 2.757	0.52381 ± 0.26427	0.54510 ± 0.25773	0.0400	(2/4) p_s_ = 0.43 p_w_ = 0.56	(4/2) p_s_ = 0.21 p_w_ = 0.16
C (Syl-c)	23	5.429 ± 3.359	0.46857 ± 0.21381	0.47312± 0.21517	0.0098	(4/2) p_s_ = 0.26 p_w_ = 0.43	(5/1) p_s_ = **0.05** p_w_ = **0.03**
D (Per)	27	6.286 ± 3.2	0.51534 ± 0.28094	0.53342 ± 0.27838	0.0174	(3/3) p_s_ = 0.53 p_w_ = 0.68	(6/0**)** p_s_ **= 0.005** p_w_ = **0.016**
F (Syl-d)	19	4.333 ± 2.160	0.49123 ± 0.21996	0.52798 ± 0.19118	0.0714	(2/3) p_s_ = 0.56 p_w_ = 0.15	(2/3) p_s_ = 0.64 p_w_ = 0.81

Syl = sylvatic, Per = peridomiciliary and Dom = domiciliary, Syl-c = conserved sylvatic, Syl-d degraded sylvatic

Gene flow among populations was estimated using F_*ST*_ pairwise comparisons. All pairwise comparisons except for three (A-D, A-F, B-D) were significantly different (Table 5). The F_*ST*_ values showed that the peridomiciliary population D is not differentiated from the domiciliary population B (F_*ST*_ = 0.00852, p<0.0001) and the sylvatic population A (F_*ST*_ = 0.00635, p<0.0001), suggesting genetic flow. The lack of differentiation between populations A and F (F_*ST*_ = -0.00078, p<00001) likely indicates that sylvatic populations belong to the same panmictic unit whereas sylvatic populations A and C are geographically related but genetically differentiated (F_*ST*_ = 0.01513; P< 0.05). The Mantel test revealed no significant isolation by distance (IBD; Z = 205.5983, r = 0.2359) among populations (See S3 File).

**Table 5:** FST pairwise population comparisons for A, B, C, D and F populations of T. brasiliensis (p-values <0.05 are bolded) from eight microsatellites loci.

	A	B	C	D	F
A	-				
B	**0.02130**	-			
C	**0.01513**	**0.02265**	-		
D	0.00635	0.00852	**0.02791**	-	
F	0.00000	**0.03253**	**0.02440**	**0.01609**	-

Genetic assignment (GeneClass2) was used to select or exclude populations as origins of individuals (Table 6). The results showed that high percentages of individuals were correctly assigned to their original population, especially for population D (93%) with lower correct assignments for populations C (80%) and F (79%). However, for sylvatic population A and domiciliary population B, 29% of the individuals were assigned to another population (Table 6).

**Table 6:** Genetic assignment (Assign) to select or exclude populations as origins of *Triatoma brasiliensis*. The percentage was computed per population and represents the probability that individuals of a given population belong to another population or are a resident of the population in which they were sampled.

	A	B	C	D	F	Assign%
A	17	1	2	2	2	71%
B	1	15	4	1	0	71%
C	3	1	20	0	0	80%
D	1	0	0	26	1	93%
F	1	1	1	1	15	79%

#### Feeding sources

The *cytb* sequencing from the DNA gut content allowed us to identify the sources of blood for approximately half of the specimens (60/126, Table 7). The limited success of PCR can be explained by the nutritional status of insects or the fact that there were multiple blood sources. In the first case, if insects were starved there would have been little blood DNA extracted from the digestive tract, making it impossible to perform the PCR. In the latter case, direct sequencing would not provide an exploitable sequence. Some sequences matching *T*. *brasiliensis* were also excluded.

**Table 7:** Infection and feeding source determination. We used two methods to determine *Trypanosoma cruzi* infection in bugs: direct examination (Dir-Inf) and PCR. Ni, is the number of positive individuals; Nf, the total number of examined individuals. Parasite characterization (TCII/TCI) was determined by PCR (see Materials and Methods). The number of feeding sources detected is indicated and their putative affiliation (putative feeding source) using BLAST searches. The Id (%) represents the identity of the sequences obtained from vector guts with those in GenBank (GB), with the GB code in the last row. Feeding sources detected in vectors infected by *T*. *cruzi* via PCR are in bold.

Pop	Dir-inf, Ni/Nt (%)	PCR-Inf, Ni/Nt (%)	TCII/TCI	Feeding sources detected (%)	Number of feeding sources	Putative feeding source	id %	Gb code	Gb species affiliation and popular name
A (Syl-c)	24/29 (83)	17/24 (70.8)	17/0	8/24 (33)	3	***Felis catus***	100	AY509646	*Felis catus* (domestic cat)
					4	***Kerodon rupestris***	96	GU136722	*Kerodon rupestris* (rock cavy)
					1	*Proceratophrys* sp.	83	KF214166	*Proceratophrys boiei* (frog)
C (Syl-c)	18/19 (95)	13/25 (52.0)	11/2	20/25 (80)	2	***Felis catus***	100	AY509646	*Felis catus*
					1	***Galea*** sp.	88	GU067492	*Galea spixii* (Spix's yellow-toothed cavy)
					15	***Keradon rupestris***	96	GU136722	*Keradon rupestris*
					1	***Thrichomys apereoides***	98	AY083338	*Thrichomys apereoides* (spiny rat)
					1	*Homo sapiens*	97	KJ8556840	*Homo sapiens* (human)
F (Syl-d)	-	5/19 (26.3)	5/0	6/19 (31.6)	3	*Galea spixii*	96	GU067492	*Galea spixii*
					2	*Capra hircus*	100	FM205715	*Capra hircus* (goat)
					1	*Felis catus*	100	AY509646	*Felis catus*
D (Per)	0/25 (0)	1/28 (3.6)	1/0	11/28 (39.3)	9	*Gallus gallus*	100	DQ512917	*Gallus gallus* (chicken)
					1	*Mabuya sp*.	79	AF280276	*Mabuya spinalis* (lizard)
					1	*Proceratophrys* sp.	78	KF214166	*Proceratophrys boiei*
H (Per)	-	0/9 (0)	0/0	8/9 (88.9)	8	*Galea spixii*	96	GU067492	*Galea spixii*
B (Dom)	0/17 (0)	0/21 (0)	0/0		4	*Capra hircus*	100	FM205715	*Capra hircus*
					2	*Gallus gallus*	100	DQ512917	*Gallus gallus*
					1	*Proceratophrys* sp.	83	JN814534	*Proceratophrys boiei*
Total	42/90 (46.5)	36/126 (28.6)	32/2	60/126 (47.6)	60				

Syl = sylvatic, Per = peridomiciliary and Dom = domiciliary, Syl-c = conserved sylvatic, Syl-d degraded sylvatic.

Overall, vertebrates identified as feeding sources of *T*. *brasiliensis* belong to ten genera distributed across four classes: Mammalia, Aves, Reptilia, and Amphibia (Table 7). Of the total number of meals identified (*N* = 60), 73% of bugs fed on mammals, 20% on birds, 5% on reptiles and 2% on amphibians. Mammals were distributed in four orders: Rodentia, Artiodactyla, Carnivora and Primate. Half of the genera were native species and half were domesticated animals or humans. The type of feeding sources varied according to the ecotope. Wild animals such as lizards, frogs and native rodents were feeding sources in both peridomiciliary and domiciliary environments. The opposite was true in sylvatic environments, where we found evidence of *T*. *brasiliensis* feeding on domestic animals such as goats (*Capra hircus*, 33%) and cats (*Felis catus*, 16%). Overall, in sylvatic environments, up to 97% of the feeding sources were mammals, and of those, 53% were rodents. The rodent feeding sources were mostly composed of the native Caviidae: *K*. *rupestris* (the *mocó* or rock cavy; 71%) and *Galea spixii* (the *preá* or Spix's yellow-toothed cavy; 16.5%).

Chickens (*Gallus gallus*) were the most prevalent (47%) feeding source in the peridomestic environment, representing 25% of feedings in domiciles. In the domiciliary ecotope, goats were the principal vertebrate feeding sources (50%), consistent with field observations that these animals circulate indoors. Eight (42%) blood sources detected in peridomiciliary areas were associated with the rodent *G*. *spixii*.

Areas A and C are located in the environmental conserved area protected by military armed forces where hunting is forbidden and used for training combats. Of the 28 feeding sources detected, 68% of bugs had fed on the native rodent *K*. *rupestris*. Nonetheless, smaller numbers of other blood sources were observed, including cats (*F*. *catus*; N = 5) and humans (*Homo sapiens*; N = 1).

### Natural infection and characterization of *Trypanosoma cruzi*

Using light microscopy, we were able to successfully analyze fecal drops from a total of 90 specimens from three populations (B, C, and D). Forty-six percent of insects were infected with *T*. *cruzi*-like parasites. The infection prevalence was highest in the sylvatic environment (83%, population A; 95%, population C).

We analyzed 126 insects using mini-exon amplification. We detected lower infection prevalence using molecular procedures than we did with direct analyses for sylvatic populations (population A: 70.8% and population C: 52%). However, we were able to detect the presence of *T*. *cruzi* parasites in population D that we did not detect with the direct method. Consistent with direct fecal laboratory observations, none of the bugs from domiciliary population B was found to be infected using the molecular approach. However, one adult female insect from domiciliary population P was positive for *T*. *cruzi*, which was not included in the Table 7 due to the low number of insects captured for this population (N = 3) (see Table 1). Each mammal feeding source (except goats) detected using *cytb* sequencing was also infected by *T*. *cruzi*, and so each should be considered potential a *T*. *cruzi* reservoir. Almost all *T*. *cruzi* were TCII; however, two samples from sylvatic population C were classified as mixed TCI and TCII lineages, exhibiting both the 300 and 350bp bands (Table 7). Notably, the positive bug from domiciliary population P was of the TCI lineage. All the TCII samples were sequenced (N = 36) resulting in only one haplotype (KT364456). The strain Tu18 (AY367125.1) showed the best hit matching (99% identity, 4e-100). A high proportion (8/19) of the bugs that fed on *K*. *rupestris* was infected with TCII, while one was a mix of TCI/TCII.

## Discussion

Intensive control measures that involved treating houses with insecticide ultimately controlled Brazil’s major *T*. *cruzi* vector, *T*. *infestans*. However, Rio Grande do Norte is one of the few Brazilian states where *T*. *infestans* had never been detected, even in places where Chagas disease was hyperendemic. These observations suggest that *T*. *brasiliensis* was always the main *T*. *cruzi* vector in this region due to its synanthropic nature [8,17]. Despite the fact that the Chagas Disease Control Program (PCDCh) mainly targeted *T*. *infestans*, domiciliary captures of some native triatomines—such as *T*. *brasiliensis—*had also decreased dramatically after PCDCh [8]. To understand the connections between elements of eco-epidemiologic channels, we need to know about the source foci for *T*. *brasiliensis* re-infestations, the natural feeding sources for triatomines and the natural infection prevalence for *T*. *cruzi*. Here, we provide a multisource approach that takes advantage of a combination of high resolution molecular tools to investigate the role of *T*. *brasiliensis* in the contemporary Chagas disease eco-epidemiology.

### *Triatoma brasiliensis* identification and genetic structure

All adult samples identified as *T*. *brasiliensis* using the taxonomic keys [7,21] were confirmed by ABGD and phylogenetic studies. Caicó is the type locality for *T*. *brasiliensis* [21], where other vector species—such as *T*. *pseudomaculata* and *T*. *petrochii—*occur in sympatry. *Triatoma pseudomaculata* is commonly found concomitantly with *T*. *brasiliensis* in peridomicilary and domicilary areas [8], whereas *T*. *petrochii* is sometimes found in rocky outcrops in the sylvatic environment [21]. In our study, we found that *T*. *petrochii* and *T*. *brasiliensis* co-occurred in some sylvatic environments and on the same rock outcrop spot. Even the adults of *T*. *petrochii* are morphologically similar to *T*. *brasiliensis*, as they were once in synonymy [21]. They are now recognized as full species and placed in the *brasiliensis* subcomplex of the *infestan*s complex [45]. This co-occurring reinforces the importance of using ABGD approaches to address the taxonomic status of *T*. *brasiliensis*, especially for nymphs, for which there are no available taxonomic keys.

Domiciliary population B had the highest haplotype diversity, revealing high genetic variability. We found that two central *cytb* haplotypes and other low-frequent haplotypes (differing in one or two nucleotide substitutions) formed a star-like haplotype network, which suggests population expansion. Even though *D* Tajima indicators were not significant, Fu’s *FS* showed that domiciliary population B and sylvatic populations C and F have more likely experienced a recent expansion event. Fu’s *FS* may be a more sensitive indicator for this event [34–35] than the Tajima *D* indicator, explaining this discrepancy. At most, four mutational steps from the central haplotype may be discerned in the network. Little structure using *cytb* haplotypes was observed, consistent with previous studies [19] for micro-scale geographic sampling.

We used F_*ST*_ to infer the gene flow between populations. A recent study [46] has shown that for inferences based on F_*ST*_ by microsatellites in diploid organisms, results are robust even with minimal sample size (N = 20). The authors stated that improving the sampling design may show better results than genotyping more than 20 samples. With our sampling design, two populations did not meet the minimum sample size (N = 17 and 19 individuals genotyped). Microsatellites applied to *T*. *brasiliensis* revealed low but significant genetic differentiation (F_*ST*_ values) between some neighboring populations, such as between sylvatic populations A and C, which are separated by less than 1 km. This differentiation may be associated with demographic events—low heterozygosity in population C might be related to bottlenecks. According to Luikart and Cornuet [47], the SSM model is robust and suitable for microsatellites. It indicated possible bottleneck events for peridomiciliary population D and for sylvatic populations A and C. Similarly, the heterozygote deficit detected in sylvatic population A using F_*IS*_ may be a result of the Walhund effect, reflecting the sampling of subpopulations with distinct allelic frequencies. It is worth noting that this sylvatic population A exhibited high indices of assignment outside of its own cluster, also suggesting that such subpopulations exist. The low variability in sylvatic population F, with both few alleles and low observed heterozygosity, may be a result of ecologic factors because it was collected in a degraded sylvatic area.

In the Bolivian Andes, observations of the genetic differentiation between sylvatic and domiciliary populations of *T*. *infestans* were used to suggest that adjacent sylvatic foci are not the source for re-colonization, but rather, survivors of insecticide treated houses [48]. In Argentina, [49] a previous study showed that *T*. *infestans* populations from communities under sustained vector control were highly structured; however, populations from communities with sporadic vector control did not show such structure. Despite the great versatility in re-infestation profiles exhibited by *T*. *dimidiata* on the Yucatan Peninsula a variety of foci for re-infestations were found, which were depending on the site and the season [50]. Each of these examples were supported by the use of microsatellites. Our results support the idea that re-infestation foci for *T*. *brasiliensis* also come from distinct sources: peridomiciliary and sylvatic areas. This finding is worrisome from a vector control perspective because sylvatic populations represent perennial and uncontrollable foci for re-infestation. Additionally, domiciliary population B and peridomiciliary population D—which came from distinct villages—were genetically related, being probably in gene flow. The direction of re-colonization of homes after insecticide spraying is likely to be from peridomiciliary to domiciliary environments [51].

### Natural feeding sources for *T*. *brasiliensis*

#### In the conserved areas

Populations A and C are located in the environmental conserved area surrounded by huge walls where hunting is strictly forbidden and domesticated animals (such as dogs, cats, goats, cattle and chicken) are removed by military guards when they eventually enter the area. The rock cavy, *K*. *rupestris*—a species of rodent also called *mocó by* locals—was the main feeding source for *T*. *brasiliensis* in the conserved sylvatic environment. However, no evidences of feeding on *K*. *rupestris* in other environments in non-preserved areas were found. This might be because *K*. *rupestris* is targeted for hunting by locals due to its relatively large size and appreciated meat, whereas *G*. *spixii* seems to be a synanthropic rodent less appreciated. A recent study [52] also detected *K*. *rupestris* and *G*. *spixii* as *T*. *brasiliensis* feeding sources in Ceara State. Indeed, the prevalence of rodent-dominant feeding sources we found supported the earlier suspicions that *T*. *brasiliensis* is primarily associated with rodents that also occur in rocky complexes [21], Domesticated cats are highly mobile and distributed throughout the city. They are also able to climb the high walls. As such, it is not surprising that we found evidence that *T*. *brasiliensis* feeds on cats within this conserved area. Because this area is used for basic combat training, the identification of human blood within these vectors highlights the risk of Chagas transmission to soldiers, particularly in the light of the high *T*. *cruzi* prevalence in these sylvatic (A and C) *T*. *brasiliensis* populations, as discussed below.

#### In non-conserved, peridomiciliary and domiciliary environments

Bugs from domiciles fed on goats, chickens and frogs, all of which were observed within domiciles during captures. The absence of human blood may be a result of movement of the bug across ecotopes: according to some authors [9,12,53,54] the triatomine dispersion capacity among ecotopes is rather neglected. Bug movement [54] must be also considered for its ability to locate feeding sources of domestic and sylvatic populations, because some domiciles are not far from sylvatic environments. Few *T*. *brasiliensis* collected from the non-conserved sylvatic environment had sufficient material in their gut contents for amplification and sequencing. However, blood sources were identified as *G*. *spixii*, goats and cats in population F, similar to species of feeding sources for peridomiciliary areas (except for the absence of chickens). It is not surprising that chicken was the major feeding source in peridomiciliary areas, as they are known to be responsible for maintaining high densities of *T*. *brasiliensis* colonies [12]. The finding that *G*. *spixii* composed half of the *T*. *brasiliensis* feeding source in peridomiciliary areas is worrisome because it indicates this rodent is no longer only sylvatic. but that it has become a highly synantropic animal that presents the potential to connect *T*. *cruzi* between the sylvatic and domiciliary cycles. Neither the frogs, *Proceratophrys* sp., nor the lizard, *Mabuya* sp., had ever been predicted as feeding sources for *T*. *brasiliensis*. Indeed, frogs and lizards are often observed in the same peridomiciliary areas as *T*. *brasiliensis* during field captures. Even though they are *T*. *cruzi* refractory [21], they may serve to maintain triatomine populations in domiciliary and peridomiciliary areas.

### *Trypanosoma cruzi* natural infection and lineages

We reported 83–95% (*T*. *cruzi*-like) and 52–70% of *T*. *cruzi* prevalence via traditional and molecular methods for sylvatic populations of *T*. *brasiliensis*, respectively. The State Health Department in Rio Grande do Norte (RN) uses direct microscopic observation of fecal drops in Chagas disease control surveillance campaigns to identify *T*. *cruzi*. Using this method, we detected higher prevalence of *T*. *cruzi* infection in sylvatic populations than using molecular methods that might be a result of misidentifying *T*. *cruzi*-like parasites for *T*. *cruzi* during direct microscopic examination. Such misidentification might be also generating some proportion of false-positive results for local control-surveillance. There is also the possibility of false negative results using molecular analyses, due to PCR inhibition and/or annealing troubles. To prevent such results, a combination of other markers should be used. However, for domiciliary and peridomiciliary areas, infected bugs were only detected via molecular methods. False negatives acquired using traditional methods of direct fecal observations might be a result of low parasitic loads in insects.

Overall infection prevalence for peridomiciliary and domiciliary environments in the entire Rio Grande do Norte state previously reported [8] was 4.3%. For both methods, our results contrast with some studies performed in Ceará State, where the *T*. *cruzi* infection prevalence in wild populations was 10.9%, with its highest peak in July [55]. On the other hand, a previous study [56] recorded higher rates in peridomiciliary (15.7%) and domiciliary (10.7%) areas than we identified in our study (≤ 3.6%). In this same area, previous studies [12] also noted 15.1% infection prevalence in Caico, without specifying the ecotope.

The division of TCI and TCII proposed by authors [26] in 1996 was based on the two major phylogenetic lineages of *T*. *cruzi*. Therefore, despite being outdated and using lower resolution to define *T*. *cruzi* genotypes according the new consensus for *T*. *cruzi* nomenclature of Discrete Typing Units (DTU) [57], our protocol exhibits an evolutionary meaning. All TCII we found based on our protocol [26] can be any of DTU II to VI, whereas TCI can be only DTU I, according to the new consensus for *T*, *cruzi* intraspecific nomenclature [57]. Indeed, by using this protocol [26], we aimed mainly to confirm *T*. *cruzi* infection and to provide clues about the diversity in the area, according to these two major parasite lineages (TCI and TCII). The TCII affiliation was confirmed by the similarity of the sequences with the Tu18 TCII strain. We found TCII prevalence (94%) similar to that found previously [58] for this same municipality. Furthermore, their study [58] had the resolution to identify also the DTU III (or TcIII), which was not targeted in our protocol. However, because TCII was prevalent in our study, further investigation is need to determine to which samples DTU corresponds, according to the new *T*. *cruzi* nomenclature. [57]. Our analysis found two (6%) TCI isolates that correspond to DTU I [57] in the sylvatic area. These findings are consistent with previous studies [58–59] conducted in the same region and confirms that sylvatic *T*. *brasiliensis* population may harbor a mixture of the two major *T*. *cruzi* lineages.

### *Trypanosma cruzi* natural infection vs feeding sources

By detecting feeding sources and natural *T*. *cruzi* infection in the same *T*. *brasiliensis* individual, we can infer potential reservoirs. Except for the peridomiciliary population, *T*. *brasiliensis* fed mainly on mammals, which might be both the vector feeding sources and the *T*. *cruzi* reservoir. Differences in natural *T*. *cruzi* infection prevalence according to the ecotope can also be explained by the differences in feeding sources. Because birds are refractory to *T*. *cruzi* infection [21], the natural infection was low in peridomiciliary areas where chickens were the dominant feeding source.

In the sylvatic environment, no birds were identified as feeding sources. Instead, rodents were the major blood source—especially *K*. *rupestris*. This rodent probably plays an important role in the epidemiological *T*. *cruzi* cycle, as we found that it was the prevalent feeding source in the same *T*. *brasiliensis* individuals that were infected by *T*. *cruzi*. This suggestion should to be critically investigated, because the infected insects may have acquired *T*. *cruzi* via any other mammal before subjected to the test.

Because cats and goats also served as feeding sources for insect vectors in sylvatic and domiciliary ecotopes, they may also link sylvatic and domiciliary *T*. *cruzi* cycles. Goats were identified recently as feeding sources for *T*. *brasiliensis* [60]; however, little is known of their role in Chagas disease epidemiology in northeastern Brazil. Here, we show a high frequency of feedings on cats in both sylvatic and domiciliary environments, including in an area with high natural *T*. *cruzi* prevalence. In Argentina [61], cats are recognized as able to maintain *T*. *cruzi* populations. Indeed, cats are a common feeding source of triatomines almost everywhere [62]. Therefore, we recommend further study on the role of cats in *T*. *cruzi* transmission in northeastern Brazil.

### Concluding remarks

Using an individual-based multisource approach, we identified potential key-links for Chagas disease eco-epidemiology between the ecotopes examined. Microsatellites showed triatomine gene flow between ecotopic populations and highlighted varied sources of domiciliary and peridomiliciary infestations, which threaten vector control efforts. The co-occurrence of two *T*. *cruzi* strains in a sylvatic *T*. *brasiliensis* population is also consistent with a link between sylvatic and domiciliary cycles. As *G*. *spixii* circulates between distinct ecotopes, the finding of feedings on this mammal reinforces [60] the need to clarify the potential of this animal to connect *T*. *cruzi* from sylvatic to domiciliary environments. The distances between sylvatic and peridomestic spots where feedings were detected on *G*. *spixii* were less than 1 km. Therefore, the identification of this rodent as vector feeding source is even more relevant. Recently some authors [63]—testing a set of *a priori* hypotheses about the drivers of site-occupancy by *T*. *brasiliensis—*showed that *T*. *brasiliensis* was strongly associated with key hosts, including rodents in peridomiciliary environments. In these findings the ecotope structure had small to negligible effects.

Our study did not characterize completely the dietary habits of *T*. *brasiliensis* because the rate of determination via direct sequencing was limited. In order to have a better representation of the host diversity, metagenomics or metabarcoding approaches using high throughput sequencing (HTS) technology is currently being developed. Additionally, to improve host assignation, the set of sequences deposited in GenBank, including for the fauna of neglected and remote sites, must likewise be improved. This limitation may have resulted in the low BLAST identity for some taxa not well studied. To exemplify this limitation, a sequence matched (with 79% of identity) an endemic lizard species (*Mabuya spinalis*) from Cape Verde Islands (Africa) [64]. This low identity may be a result of feedings on a related reptile species because there are records of occurrence of some other *Mabuya* sp. in northeastern Brazil [65]. Therefore, to improve host assignation, it will require the mobilization of taxonomists specialized on vertebrates and the constitution of a broader set of GenBank reference sequences.

Because Chagas disease may be also transmitted through contact with hunted animals, as is suggested for the Amazon region in Brazil [66], caution is necessary when handling these animals. Moreover, educational campaigns should address this issue. We recommend avoiding creating suitable habitats for *G*. *spixx* in peridomiciles [see 63] because it may connect sylvatic and domestic *T*. *cruzi* cycles.

Chagas disease control is complex by nature due to an overlap of sylvatic, peridomestic and domestic cycles. However, the use of integrative approaches, such as population genetics to estimate gene flow between habitats combined with ecological data in a landscape genetics perspective [67–68] as well as an individual-based approach, linking vector population genetics to host and parasite identification, may help to better understand the eco-epidemiology of Chagas disease.

## Supporting Information

S1 File Bayesian inference consensus tree of the *Triatoma brasiliensis cytb* gene. Additional sequences used for the phylogenetic reconstruction were downloaded from GenBank (GB) and were identified by their GB code after the (sub)specific taxonomic definition. *Triatoma sherlocki* was defined as the outgroup. Values in the nodes indicate Bayesian posterior probabilities. All of our samples fit in the *T*. *brasiliensis* cluster defined by Costa et al. (2013).
(JPG)

S2 File Variable positions in the 467 bp of the *cytb* gene for the 28 haplotypes of *T*. *brasiliensis* collected in the study according to sites where they were collected. (PDF)

S3 File Each spot represents the genetic differentiation [F_ST_/(1−F_ST_)] of one unique pair of *Triatoma brasiliensis* populations correlated with linear distance. (JPG)
