# Supplementary figures and images for: Molecular Individual-Based Approach on Triatoma brasiliensis: Inferences on Triatomine Foci, Trypanosoma cruzi Natural Infection Prevalence, Parasite Diversity and Feeding Sources
Source: PLoS Negl Trop Dis. 2016 Feb 18;10(2):e0004447. doi: 10.1371/journal.pntd.0004447 (PMC4758651; doi:10.1371/journal.pntd.0004447)

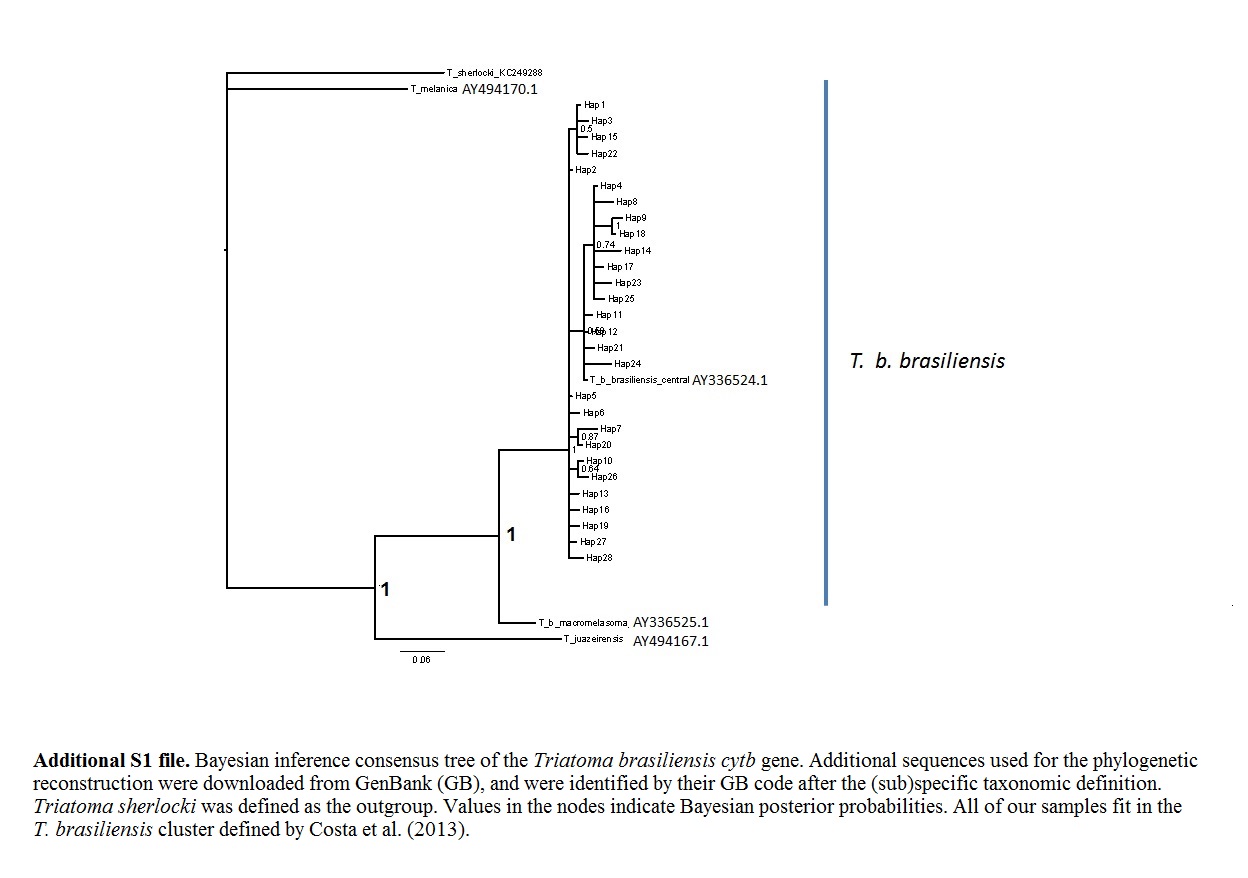

Supplement: S1 File — Additional sequences used for the phylogenetic reconstruction were downloaded from GenBank (GB) and were identified by their GB code after the (sub)specific taxonomic definition. Triatoma sherlocki was defined as the outgroup. Values in the nodes indicate Bayesian posterior probabilities. All of our samples fit in the T. brasiliensis cluster defined by Costa et al. (2013). (JPG) [file pntd.0004447.s001.jpg]

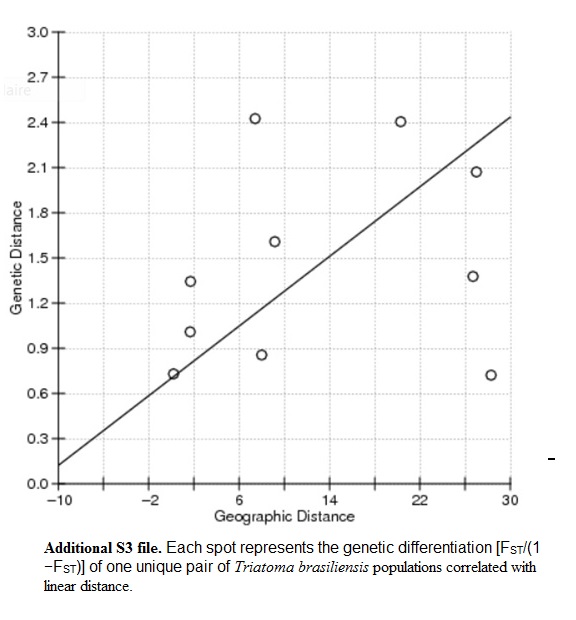

Supplement: S3 File — (JPG) [file pntd.0004447.s003.jpg]
