# Supplementary material for: Molecular Individual-Based Approach on Triatoma brasiliensis: Inferences on Triatomine Foci, Trypanosoma cruzi Natural Infection Prevalence, Parasite Diversity and Feeding Sources
Source: PLoS Negl Trop Dis. 2016 Feb 18;10(2):e0004447. doi: 10.1371/journal.pntd.0004447 (PMC4758651; doi:10.1371/journal.pntd.0004447)
Supplement: S2 File — (PDF) [file pntd.0004447.s002.pdf]

Variable positions in the 467 bp of the cytb gene for the 28 haplotypes of *T. brasiliensis* collected in the study according to sites where they were collected

Hap	Nucleotide sites																																	Populations					Hap number	Hap frequency	GeneBank code
							1	1	1	1	1	2	2	2	2	2	2	2	3	3	3	3	3	3	3	3	3	3	3	4	4	4	4								
			1	1	2	8	1	2	4	8	8	1	3	5	5	6	6	9	2	2	3	4	5	5	5	6	8	8	9	0	2	3	3	5								
	5	6	3	6	9	3	0	1	0	2	5	6	6	1	4	0	3	7	0	6	2	4	0	4	8	1	0	5	8	4	2	2	3	2	B	D	A	C	F	total	%	
**Hap_1**	A	A	T	T	G	G	T	T	A	T	T	C	C	T	A	C	A	A	A	T	T	G	A	G	T	C	T	T	G	A	A	A	T	A			**3**	**1**		**4**	3.77	KT336389
**Hap_2**	G	.	.	.	.	.	.	.	.	.	.	.	.	C	.	.	.	.	.	.	.	.	.	.	.	.	.	.	.	.	.	.	.	.	**4**	**2**	**7**	**6**	**2**	**21**	19.81	KT336390
Hap_3	.	.	.	.	.	.	.	.	.	.	.	.	.	.	.	.	.	.	.	.	.	.	.	.	.	.	.	.	.	.	G	.	.	.			1			1	0.94	KT336391
**Hap_4**	G	.	.	.	.	.	.	.	G	.	.	.	.	.	.	.	.	.	.	C	.	.	.	.	.	.	.	.	.	.	.	.	.	.			**4**	**1**		**5**	4.72	KT336392
**Hap_5**	G	.	.	.	.	.	.	.	.	.	.	.	.	.	.	.	.	.	.	.	.	.	.	.	.	.	.	.	.	.	.	.	.	.	**2**	**12**	**3**	**6**	**5**	**28**	26.42	KT336393
Hap_6	G	.	.	.	.	A	.	.	.	.	.	.	.	.	.	.	.	.	.	.	.	.	.	.	.	.	.	.	.	.	.	.	.	.			1			1	0.94	KT336394
**Hap_7**	G	.	.	.	.	.	.	C	.	.	.	.	.	.	T	.	.	.	.	.	C	.	.	.	.	.	.	.	.	.	.	.	.	.			**1**	**1**		**2**	1.89	KT336395
**Hap_8**	G	.	.	.	.	.	.	.	G	.	.	.	.	.	.	.	.	G	.	C	.	.	.	.	.	.	.	.	.	G	.	.	.	.			**2**	**1**		**3**	2.83	KT336396
**Hap_9**	.	.	.	.	.	.	C	.	G	.	.	.	.	.	.	.	.	.	.	C	.	.	.	.	.	.	.	.	A	.	.	.	.	.	**2**	**1**				**3**	2.83	KT336397
Hap_10	G	.	.	C	.	.	.	.	.	.	.	.	.	C	.	.	.	.	.	.	.	.	.	.	.	.	.	.	.	.	.	.	.	.	1					1	0.94	KT336398
Hap_11	G	.	.	.	A	.	.	.	G	.	.	.	.	.	.	.	.	.	.	.	.	.	.	.	.	.	.	.	.	.	.	.	.	.	3					3	2.83	KT336399
**Hap_12**	G	.	.	.	.	.	.	.	G	.	.	.	.	.	.	.	.	.	.	.	.	.	.	.	.	.	.	.	.	.	.	.	.	.	**1**			**1**	**4**	**6**	5.66	KT336400
Hap_13	G	.	.	.	.	.	.	.	.	.	.	.	.	.	.	.	.	.	.	.	.	.	.	.	.	.	C	.	.	.	.	.	.	.	1					1	0.94	KT336401
Hap_14	G	.	G	.	.	.	.	.	G	C	C	.	.	.	.	.	.	.	.	C	.	.	.	.	.	.	.	.	.	.	.	.	.	.	1					1	0.94	KT336402
Hap_15	.	.	.	.	.	.	.	.	.	.	.	.	.	.	.	.	G	.	.	.	.	.	.	.	.	.	.	.	.	.	.	.	.	.	1					1	0.94	KT336403
Hap_16	G	.	.	.	.	.	.	.	.	.	.	.	.	C	.	.	.	.	.	.	.	.	.	.	.	.	.	.	.	.	.	T	.	.	1					1	0.94	KT336404
**Hap_17**	G	.	.	.	.	.	.	.	G	.	.	.	.	.	.	.	.	.	.	C	.	.	G	.	.	.	.	.	.	.	.	.	.	.		**1**		**3**	**2**	**6**	5.66	KT336405
**Hap_18**	.	.	.	.	.	.	C	.	G	.	.	.	.	.	.	.	.	.	.	C	.	.	.	.	.	.	.	.	.	.	.	.	.	.				**1**	**2**	**3**	2.83	KT336406
Hap_19	G	.	.	.	.	.	.	.	.	.	.	.	.	C	.	.	.	.	.	.	.	.	.	.	.	T	.	.	.	.	.	.	.	.				1		1	0.94	KT336407
**Hap_20**	G	.	.	.	.	.	.	.	.	.	.	.	.	.	.	.	.	.	.	.	C	.	.	.	.	.	.	.	.	.	.	.	.	.		**1**		**1**		**2**	1.89	KT336408
Hap_21	G	.	.	.	.	.	.	.	G	.	.	.	T	.	.	.	.	.	.	.	.	.	.	.	.	.	.	.	.	.	.	.	.	.		2				2	1.89	KT336409
Hap_22	.	.	.	.	.	.	.	.	.	.	.	.	.	.	.	.	.	.	G	.	.	.	.	.	.	.	.	.	.	.	.	.	.	.		5				5	4.72	KT336410
Hap_23	G	.	.	.	.	.	.	.	G	.	.	.	.	.	.	.	.	.	.	C	.	A	.	.	C	.	.	.	.	.	.	.	.	.		2				2	1.89	KT336411
Hap_24	G	.	.	.	.	.	.	.	G	.	.	.	.	.	.	T	.	.	.	.	.	A	.	.	.	.	.	.	.	.	.	.	.	G		1				1	0.94	KT336412
Hap_25	G	G	.	.	.	.	.	.	G	.	.	.	.	.	.	.	.	.	.	C	.	.	.	.	.	.	.	.	.	.	.	.	.	.					1	1	0.94	KT336413
Hap_26	G	.	.	C	A	.	.	.	.	.	.	.	.	C	.	.	.	.	.	.	.	.	.	.	.	.	.	.	.	.	.	.	.	.					1	1	0.94	KT336414
Hap_27	.	.	.	.	.	.	.	.	.	.	.	.	.	C	.	.	.	.	.	.	.	.	.	.	.	.	.	.	.	.	.	.	.	.					1	1	0.94	KT336415
Hap_28	G	.	.	.	A	.	.	.	.	.	.	.	.	C	.	.	.	.	.	.	.	.	.	.	.	.	.	.	.	G	.	.	.	.					1	1	0.94	KT336416
Total																																			17	27	22	23	19	108	100.00	
